# Health Disparities and Toxicant Exposure of Akwesasne Mohawk Young Adults: A Partnership Approach to Research

**DOI:** 10.1289/ehp.7914

**Published:** 2005-07-18

**Authors:** Lawrence M. Schell, Julia Ravenscroft, Maxine Cole, Agnes Jacobs, Joan Newman, Akwesasne Task Force on the Environment

**Affiliations:** 1 Department of Epidemiology, University at Albany, State University of New York, USA; 2 Department of Anthropology, University at Albany, State University of New York, USA; 3 First Environment Research Projects, Akwesasne Mohawk Nation, Akwesasne, New York, USA; 4 Department of Educational Psychology and Statistics, University at Albany, State University of New York, USA; 5 Akwesasne Mohawk Nation, Akwesasne, New York, USA

**Keywords:** adolescents, Akwesasne Mohawk Nation, community-based participatory research, health disparities, Native American, partnership research, polychlorinated biphenyls

## Abstract

In this article we describe a research partnership between the Akwesasne Mohawk Nation and scientists at the University at Albany, State University of New York, initiated to address community and scientific concerns regarding environmental contamination and its health consequences (thyroid hormone function, social adjustment, and school functioning). The investigation focuses on cultural inputs into health disparities. It employs a risk-focusing model of biocultural interaction: behaviors expressing cultural identity and values allocate or focus risk, in this instance the risk of toxicant exposure, which alters health status through the effects of toxicants. As culturally based behaviors and activities fulfill a key role in the model, accurate assessment of subtle cultural and behavioral variables is required and best accomplished through integration of local expert knowledge from the community. As a partnership project, the investigation recognizes the cultural and socioeconomic impacts of research in small communities beyond the production of scientific knowledge. The components of sustainable partnerships are discussed, including strategies that helped promote equity between the partners such as hiring community members as key personnel, integrating local expertise into research design, and developing a local Community Outreach and Education Program. Although challenges arose during the design and implementation of the research project, a collaborative approach has benefited the community and facilitated research.

There is considerable concern about the possible effects of endocrine-disrupting compounds such as polychlorinated biphenyls (PCBs) on the development of thyroid function (Brouwer et al. 1998; Osius et al. 1999; Persky et al. 2001; Ribas-Fito et al. 2003) and neurobehavioral maturation (Guo et al. 1994; Jacobson et al. 1990; Schantz et al. 2003). Risk of exposure to environmental contaminants such as PCBs is not an individual choice but is related to larger political and economic factors. Minority communities are often at special risk of exposure, as they are more often affected by toxic landfills, incinerators, dumping, mining, and other environmentally damaging activities (Akwesasne Notes 1993; Bryant et al. 1992; Chavis et al. 1983; Commission for Racial Justice, United Church of Christ 1987; Mohai and Bryant 1992; Schell and Czerwinski 1998). Native Americans especially suffer from a combination of these risk factors as they strive to maintain cultural identity, are often economically disadvantaged, and are perceived by mainstream society as ethnically distinct.

The Mohawk Nation at Akwesasne, New York, “the land where the partridge drums” (LaDuke 1999, p 11), has shared a disproportionate amount of environmental injustice since the construction of the St. Lawrence Seaway and the St. Lawrence–FDR Power Project in the 1950s. Cheap hydroelectric power led to the development of several major industries directly upstream, upwind, and upgradient from the community. The industrial sites have contaminated the St. Lawrence with PCBs (Ecology and Environment, Inc. 1992; RMT, Inc. 1986; Woodward-Clyde Associates 1991), and Akwesasne now sits directly adjacent to a National Priority Superfund Site while two New York State Superfund sites are nearby and immediately upriver [U.S. Environmental Protection Agency (EPA) 1984]. Some local species of fish, birds, amphibians, and mammals have PCB levels that exceed the U.S. Food and Drug Administration’s tolerance limits for human consumption (Forti et al. 1995;Lacetti 1993; Sloan and Jock 1990). Akwesasne is a potential candidate for designation as an Environmental Justice Community (EJC) by the U.S. EPA because of elevated PCB levels in adjacent lands and traversing waterways, and the potential for inhalation exposure from volatized PCB particulates.

In this community, diet, particularly consumption of fish and other aquatic animals, is an important route of exposure*.* Another route for newborns and infants is via breast milk (Fitzgerald et al. 2001, 1998, 1992). PCBs are lipophilic, and, consequently, a mother’s PCB burden is passed on through breastfeeding. The passage of PCB body burdens across generations highlights the importance of exploring the household and familial contexts that pattern exposure risk. From a public health perspective, a solution is to implement educational intervention programs that discourage community residents from engaging in activities that might increase their exposure to local contaminants. However, Native Americans “are unique cultural and political groups who have very distinct environmental problems” (U.S. EPA 1992), as risk of exposure to environmental contaminants is embedded within active participation in the culture of this community and, indeed, within cultural survival itself.

Thus, the Mohawk community at Akwesasne has found itself with two alternatives, neither of which is fully acceptable to the community. The first is to continue dietary and cultural practices that increase exposure to environmental contaminants; this is, of course, not an option for many community members because of the health risks to adults, children, and generations to come. The second is to ask community members to avoid dietary and cultural practices related to exposure. The Mohawk community has followed the recommendations of tribal and state fish advisories implemented in the 1980s and early 1990s, and the levels of PCBs in breast milk and serum have fallen (Fitzgerald et al. 1995, 1998, 2004). At first glance this history may appear to be a public health policy success story, but this interpretation does not consider the specific cultural context and implications. For many Native communities, subsistence-based activities are part of the surviving traditional culture and identity. A fundamental component of Mohawk identity is that the ties linking individuals, families, and groups to specific locations and land have symbolic and sacred meaning. For the Mohawk community, being asked to avoid activities that reaffirm Mohawk identity is not a solution to this problem but a bigger problem in and of itself (Arquette et al. 2002). Because the cultural integrity and continuation of the Mohawk people is at stake, before any intervention process begins, those activities that truly place individuals at risk should be identified [Akwesasne Task Force on the Environment (ATFE) 1997] and placed in the context of Native rather than Western, economic, environmental, or social priorities (Arquette et al. 2002).

The goal of the present research is to identify adverse health effects among older adolescents and young adults stemming from exposure to local pollutants through behaviors that express cultural values and affirm cultural identity. If we can determine the behavioral pathways to health effects of interest, it may be possible for the community to continue activities that contribute to national and cultural sovereignty while not harming well-being. The health outcomes of interest are effects on thyroid function and measures of school performance and community adjustment. These outcomes were chosen because they represent the intersection of prior scientific investigations on PCBs and child development (Brouwer et al. 1998; Guo et al. 1994; Jacobson et al. 1990; Osius et al. 1999; Persky et al. 2001; Ribas-Fito et al. 2003; Schantz et al. 2003; Schell et al. 2002, 2004; Winneke G, Bucholski A, Heinzow B, Kramer U, Plabmann S, Schmidt E, et al., unpublished data) and community concerns for the social, spiritual, and physical well-being of their youth. In this article we describe the community–academic partnership that developed and implemented a program of research to investigate the impact of environmental contaminants on the health of young adults.

## Conceptual Framework—Scientific Model and the Need for Indigenous Knowledge

This research is based on the risk-focusing model (Schell 1997). The model was developed during a study of multigenerational effects of lead exposure. The model described how disability from lead exposure in early life leads to many outcomes, including reduced cognitive performance, reduced educational opportunity, reduced opportunities for employment, and greater chances of residing in an area of higher lead exposure in which another generation is exposed (Schell 1992). The model acknowledges that risk in stratified societies is apportioned according to the social and biological characteristics of individuals. Risk is said to be “focused” because several different types of risk commonly occur simultaneously in individuals with shared characteristics, and the risks are compounded over generations. None of the events in such a sequence are the result of individual choices, but each results from larger economic and social forces.

**Figure 1:**
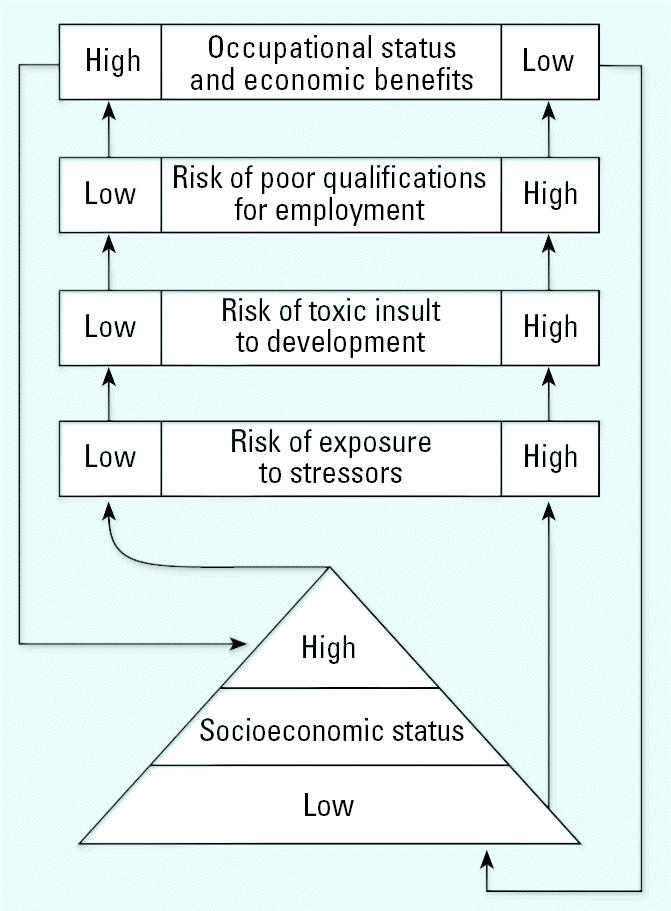
Risk-focusing model.

The risk-focusing model is a complement to models of resource allocation common in health disparities research in which resources are allocated on the basis of socioeconomic characteristics (Schell 1997). Risk focusing also recognizes that risk may be allocated on the basis of the socioeconomic characteristics that are themselves the consequences of previous exposures in one or several generations. A general model first relates social position to risk of exposures (environmental, occupational, etc.), then exposures to disabilities and suboptimal health, and, finally, disabilities to social position and further stratification (Figure 1). Any social group, whether defined in terms of biological characteristics or ethnicity or occupation, can experience suboptimal health and disabilities through exposure to environmental pollutants and then reduced opportunities for socioeconomic rewards because of poorer health.

Studies of environmental crises and disasters, such as the Exxon Valdez oil spill (Palinkas et al. 1992) and other similar incidents (Curtis 1992; Grinde and Johansen 1995; Harris and Harper 1997; Hild 1998), suggest that Native groups might be disproportionately affected by environmental pollution because of subsistence systems and a cultural ethos that involve greater contact with the physical environment. The meaning of “land,” and the environmental contamination of that land, has a spiritual significance that not only contributes to individual health but also affects identity and well-being at a group level (Arquette et al. 2002; Ransom and Ettenger 2001). Environmental contamination not only disrupts sacred ties and connections to place but also disrupts the practice of many activities such as consuming locally caught fish, trapping, hunting, gardening, and gathering materials for basket-making that express and reaffirm Mohawk identity and culture. These activities have important cultural and spiritual meaning but place individuals in direct contact with local contaminants that may increase exposure. Thus, contamination of the local environment is experienced by the community as a threat to cultural identity because avoidance of PCBs involves the inability to practice activities that are important to the Mohawk way of life and connection to the land.

**Figure 2:**
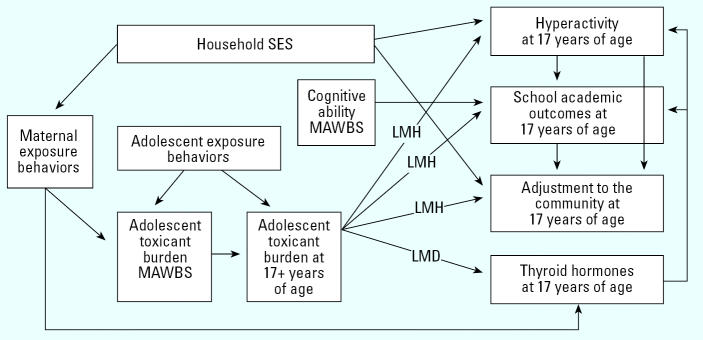
Research design. Model of primary relationships presents a diagram of relationships among primary variables in YAWBS but also includes some variables from MAWBS to indicate some longitudinal components. SES, socioeconomic status; YAWBS, Young Adult Well-Being Study. Susceptibility factors are indicated by letters: D, diet; H, mercury; L, lead; M, metabolism. For clarity, some covariates [e.g*.*, non-focal toxicants such as hexachlorobenzene, mirex, and dichlorophenyldichloroethylene (DDE)] are not depicted but will be examined.

In applying a risk-focusing model to Akwesasne (Figure 2), socioeconomic position now refers to behaviors that affirm Mohawk identity as well as usual components of socioeconomic status. The model allows us to capture the transgenerational pathway of exposure to PCBs via prenatal and lactational pathways that stem from maternal exposure. The model also includes susceptibility factors that may be allocators of risk, such as age, sex, differences in metabolism and storage of PCBs, and concurrent exposure to other toxicants. Data on toxicant levels and growth from a previous study (Gallo et al. 2005; Schell et al. 2003) of the same cohort when members were 10–17 years of age [the Mohawk Adolescent Well-Being Study (MAWBS), 1995–2000] are integrated to provide additional context, time depth, and control variables.

In applying the model to the current study, household socioeconomic position is related to possible “exposure behaviors” identified by Mohawk community members, such as fishing, hunting, picking berries and herbs, and cultivating gardens, that may allocate exposure. Because diet is another possible exposure pathway, extensive data are collected on adolescent consumption of locally caught fish and game over the last five years. A history of maternal local food consumption before and during her pregnancy with the participating adolescent is also collected to assess cross-generational dietary patterns. The ability of these behaviors to increase exposure of mothers and children to contaminants is assessed at two time points by determining the levels of PCBs and other contaminants in serum of the adolescents between 10 and 17 years of age while participating in the MAWBS and during the current project, the Young Adult Well-Being Study (YAWBS).

The primary health outcomes of interest are thyroid hormone function and measures of neurobehavioral maturation that pertain to school performance and community adjustment. The model considers pathways by which toxicants may affect such important domains directly and indirectly through hyperactivity or/and alterations in thyroid function. These relationships are assessed in individuals at 17 years of age.

Measures of thyroid function (levels of thyrotropin, total and free triiodithyronine, and thyroxine) are conducted following standard laboratory procedures. School functioning and adjustment is assessed in terms of grades, standardized test scores from state or provincial testing, and indicators of disciplinary action and school absences. Teachers complete two rating scales to describe the adolescent’s school behavior. The first of these is the Conners’ Rating Scales–Revised: Teacher Form (Conners 1997). Because of evidence from previous studies of problems of attention and activity level associated with PCB exposure, the teachers are also asked to complete a second rating scale, the Attention Deficit Disorders Evaluation Scale (McCarney 1995). Those 17-year-old individuals not attending school are interviewed to determine their age at dropping out of school, current employment status, membership in community organizations, and delinquency (any arrests or probation). Attendance records for the last year of school are also sought. Information about community membership, involvement, and delinquency is obtained from those adolescents who are still attending school.

The model tests pathways between cultural values, actualizing behavior, exposure, and health effects. It involves variables that differ considerably in their degree of standardization. Outcomes are measured with standard techniques (i.e., measures of toxicant exposure, thyroid function, standardized tests of hyper-activity), while measures of proximate and distal causes of these outcomes (values and culturally expressive behaviors) are tailored to the specific cultural context to obtain a detailed sociocultural analysis. Clearly, the accurate and reliable measurement of causal variables in the model depends on knowledge of the community—knowledge that may be provided best by the community itself.

## Rationale for a Partnership Study

To address community concerns, a partnership developed between academic researchers at the University at Albany and the Akwesasne Mohawk Nation. Optimal research partnerships with Native communities should reflect that each Native community is a unique entity with specific historical, social, political, economic, and cultural contexts (Holkup et al. 2004; Ransom and Ettenger 2001) and challenges. There is no one research “template” or strategy to apply across all Native peoples. Because the Akwesasne community is burdened by environmental contamination and exposure to toxicants, any partnership with researchers must be aimed at resolving this burden.

Past research at Akwesasne, as in many Native communities, has often proceeded in a manner that benefited those performing the research, in the form of academic advancement and grant support, rather than benefiting the community itself (Arquette et al. 2002; Ransom and Ettenger 2001; Schell and Tarbell 1998). Often, research at Akwesasne progressed with research agendas dictated by researchers with little or no opportunities for community input, and there was no clear presentation of any results or findings by scientists to the community when the research was finished (Schell and Tarbell 1998).

In the mid-1980s, as the community faced an ongoing environmental crisis caused by industrial pollution, the need to become active in the research occurring within Akwesasne territory became evident to many in the community. In response, the Akwesasne Task Force on the Environment (ATFE), a community-based organization, was founded to “conserve, preserve, and protect the natural and cultural resources within the territory of Akwesasne” (ATFE 1996; ATFE and Research Advisory Committee 1997). In 1995 the ATFE established a subcommittee, the research advisory committee (RAC), to review and comment on proposals for research to be conducted at Akwesasne. The RAC developed and published a research protocol (ATFE 1996; ATFE and Research Advisory Committee 1997) that included a set of research requirements to help outside academic researchers become collaborative partners to benefit both academia and the community.

## Conceptual Framework of the Partnership: The Akwesasne Research Protocol

The RAC developed three guiding principles for research based on the Haudenosaunee (“People of the Longhouse,” of which the Mohawk Nation is one of six Iroquois Nations) principles of peace, good mind, and strength*.* It is the emerging behaviors that flow from these guiding principles that serve to inform the research process and “channel the inherent good will of humans to work toward peace, justice, and unity to prevent the abuse of human beings and mother earth” (ATFE and Research Advisory Committee 1997, p 95). The protocol also provides specific guidelines to researchers regarding community expectations relating to full disclosure to the community of the proposed study’s methods and goals, funding sources, ongoing review of the research process and opportunities for community feedback, benefits to the community, and capacity-building through local training and hiring. By following such guidelines, a collaboration between researchers and the community is built that is based upon respect, equity, and empowerment, and which produces what the community calls “a good research agreement.”

Equity for the community can include the provision of monies to hire community researchers and/or an administration fee to support the infrastructure of a community organization. Empowerment includes not only training community people to conduct research but also that the university partners provide expertise regarding environmental contaminants and possible adverse health effects. Health effects research is valuable for the knowledge it produces, but to attain the full value of this knowledge, it must be applied in the development of health prevention strategies, new policies, and amendments to existing policy and legislation. Within the university, areas of expertise exist that can provide the community with a broad base of information and support to pursue policy change.

In addition to equity and empowerment, to develop a good research agreement the researchers and the community must generate respect for each other. Respect is generated by understanding each others’ social, political, and cultural structures (Harrison 2001; Holkup et al. 2004; O’Fallon and Dearry 2002). Examples of respect are good communication strategies that work for both partners, cultural sensitivity training for the researchers, and community awareness presentations that are clarified and questioned by each partner. Ultimately, if the need arises, consensus and mediation processes can be used to develop procedures that can be honored by both the researchers and the community.

## Roles and Responsibilities: Components of a Sustainable Partnership

The Akwesasne model recognizes that research has profound effects on any community and seeks to channel these influences to produce benefits for the community while also respecting the researchers’ needs. The economic benefits for communities are valued in the partnership, but researchers may not perceive the benefits of research projects beyond the new knowledge produced. Research for career advancement without concern for community impacts is termed “stepping-stone research” by Akwesasne partners, because it uses the community only as a stepping-stone for career advancement. It is the antithesis of partnership research, as it does not empower, respect, or promote equity in the community. Therefore, the first step in our partnership was recognizing the mutual and individual benefits of research and consciously apportioning group effort toward achieving each partner’s goals. The honesty and candor required for this activity was a trust-building exercise.

### Taking time to learn about one another.

The current team became acquainted with the Akwesasne community when planning MAWBS, which was part of a Superfund Basic Research Program grant (1995–2000). MAWBS was conducted after a process of personnel and project vetting by community members and organizations in which possible projects were described and scientists were introduced to community members interested in environmental and health problems. The conversations between researchers and community members, leaders, and organizations gave researchers the opportunity to hear the specific concerns of the community. At this point, three different driving forces were recognized: *a*) the research questions and activities the sponsor would support, *b*) what the community was interested in learning and the activities the community would support or allow, and *c*) the areas of academic researchers’ expertise. Community members and academic researchers then determined the fit among these three forces, and they agreed to submit a grant proposal.

It is also beneficial for academic researchers to attempt to understand the community’s history, politics, and culture (Harrison 2001; Holkup et al. 2004; Minkler 2004). Community members at Akwesasne mentioned frequently that when outside scientists made no attempt to at least learn a little about local culture, it appeared that the scientists were either disinterested or lacked respect for the community. At the beginning of the project, cultural sensitivity training sessions were scheduled to allow researchers to gain greater understanding of Mohawk culture and thereby produce the relationship most beneficial to both partners. In an effort to build awareness about the community, academic researchers also attended community events and assisted the community in various activities, with the intent of having a presence within the community. The ultimate goal was to build a trusting relationship for mutual benefit.

Partnering with a community before the research project is launched is best to devise a mutually beneficial research investigation (Israel et al. 1998, 2001; O’Fallon and Dearry 2002), but paying for community members’ input maybe difficult. While compensation for community input is ethical, grantors typically will not allow payments for work conducted before the funded project period begins. If other sources of compensation are not available, the community partners should be informed of the entrepreneurial and risky nature of research applications in order to budget their time and involvement.

### Understanding styles of decision making.

Researchers and the Akwesasne community differ in their styles of decision making. Among scientists the general model of decision-making is a balance of majority rule and deferring to an expert opinion, then moving on as quickly as possible to the next decision to be made. The Mohawk style of decision making is based on consensus building and everyone having the opportunity to speak. In Native communities already dealing with many factors that promote divisiveness, group solidarity is an important principle; therefore, a research project that promotes dissension (even inadvertently) will be harmful. Native governments and organizations often need time to consider the proposed project and make a decision about participation. Although it is not the role of the researcher to solve longstanding problems in Native communities, there is a need to appreciate the resources, time, and commitment that are necessary to promote community consensus.

The communication needed for reaching a consensus usually entails a longer process of decision-making. For scientists, consensus building can appear to be an exhaustive and time-consuming process. In a society where time means money and the production of new knowledge is routinely weighed against its cost, extending this process may be viewed negatively. However, obtaining community input and consensus was crucial to the success of research at Akwesasne. It enabled us to identify problems appropriately, formulate research questions, select appropriate methodologies, identify evaluation strategies, and select effective means of dissemination and education. Thus, we as researchers learned to adjust our work schedule and to build in time for this process.

### The role of community partners in the development of research design and protocols.

Native communities/governments have a primary responsibility to ensure that their citizens who participate in research do not have their human rights exploited and that protections are in place to guard participants’ health and safety. The ATFE has managed the safeguarding of human rights through the development of culturally based research guidelines. The guidelines require submitting research proposals to the RAC for review to ensure that external researchers and organizations adhere strictly to the community’s established research guidelines, particularly to how the community as a whole should be approached for review of proposed studies. When individual community members are approached regarding proposed studies, individuals cannot ensure that their or the community’s best interests will be served. The RAC was created to ensure that the rights of the community are addressed, and, consequently, that the rights of individuals are preserved, because individual rights are nested within protections afforded the community.

### Promoting equitable benefits.

One key principle of our partnership is that there should be benefits to both the community and the scientists and that these benefits serve one another. The prime community benefit is greater knowledge that enables choosing the most effective steps to alleviate the community’s toxicant burden. However, other benefits can include increased capacity in community leadership and in research performance, and these benefits can be long lasting. To build leadership capacity, community members are included as key project personnel. This practice also promotes equitable participation and influence by community partners in the project and creates structured lines of communication. Additional benefits accrue when community members are trained to do the research so that the training stays in the community (Israel et al. 2001).

These actions have direct benefits to the project as well. In our project, individually and through the RAC, community partners have played an integral role in research design and development of research protocols, including questionnaires and instruments. The RAC reviewed the grant application with the community project staff members and provided feedback regarding the cultural appropriateness and acceptability of interview questions and study procedures, other more effective ways to ask certain questions, suggestions on how to streamline data collection to minimize the burden on the participant, and additional questions that should be asked that were not readily apparent to researchers. For example, we wanted to ask about the young adults’ consumption of local foods. Through working closely with the community we were able to identify the full range of locally trapped, hunted, fished, and grown food sources; issues of seasonality that would affect data collection; and the most appropriate time units in which to collect the data.

Community members hired as part of the project were trained in the skills necessary to carry out the project, such as anthropometric measurement, food frequency assessment, in-person interviews, and data coordination. Local community members served as the experts on how such methods might best be implemented in their particular community, and their expertise and knowledge was integrated into data collection strategies. The community-based personnel collecting data provided continuous feedback to researchers in Albany regarding the utility of the instruments. When encountering problems with specific questions or an instrument, data collectors were able to make insightful suggestions on how to restructure the question or instrument to collect the data of interest in a way that was understandable and acceptable to the participant.

The community-based staff member who recruited participants received training in human “subjects” protocols, and she also capitalized on her intimate knowledge of the community, specifically of the children, to facilitate recruitment. This researcher had to consider that, in this Mohawk community, there was a specific protocol to follow regarding involvement of children in any activity. In a matrilineal society such as the Akwesasne, the mothers’ responsibilities are to nurture and care for their children; therefore, the researcher routinely approached the mother first, as the primary caregiver, then the father second. In addition community members on the research team knew appropriate avenues to publicize the study and were available to discuss the project one-on-one at informal community events.

Other complexities of this community may not be familiar to outside researchers. The Akwesasne community has a traditional government and two imposed elected governments because it straddles the U.S./Canadian border. Many parents are employed by these governments, and their children attend schools following varying schedules. Data collection appointments had to be scheduled without interfering with tribal ceremonies or Canadian and U.S. school and work holidays. The community researcher also accommodated the adolescents’ school and extracurricular activities within their own schedules. For example, because fasting blood specimens were needed, the time the families felt was most convenient for venipunctures was at home before their children left for school. The researcher then took the individual to school if necessary. To make participants comfortable and improve retention, interviews and measures of height and weight also were conducted in the home. However, other accommodations depended on knowing details of local norms. For example, a standard research practice is to maintain friendly eye contact with the participant during the interview, but at the Akwesasne community, researchers noted that minimal eye contact made participants in this young age group more comfortable. In short, the knowledge held by local project personnel served as the basis for the successful recruitment and retention of participants. In the on-going study our recruitment rate is currently 65%, a highly successful rate for a follow-up study of a hard-to-reach age-group.

In the data analysis and report writing stage, community partners contribute to interpreting variables in analyses (especially variables representing social constructs), because the community has interpretive insights that may not be apparent to the academic partners. Thus, valuing and integrating local expert knowledge enables the community to become active participants in the research process and improves design, recruitment, data collection, and interpretation of results (Holkup et al. 2004; Israel et al. 2001; Stevens and Hall 1998).

Local expert knowledge is also essential for the creation of an effective Community Outreach and Education Program (COEP). The COEP was developed by the community, with researchers’ influence limited to the interpretation of the sponsor’s requirements. A community member from Akwesasne who had experience working on the previous project (MAWBS) invested personal time to write the COEP component for the current research project (YAWBS). That individual was aware of the RAC/ATFE research guidelines and used these as a foundation to prepare the proposal. Accordingly, the individual consulted with other community members and gained feedback at the monthly ATFE meeting, a process that identified which outreach programs would be of most use to the community. After funding was awarded, this individual was hired as the director for the COEP. Community leadership of the COEP acknowledges the community’s agenda and enables community members at Akwesasne to prioritize activities for support with grant funds. One of the welcomed COEP activities is sponsorship of a bilingual local radio show during which environmental messages are conveyed in the Mohawk language Kahniakeha.

Another strategy that helps to maintain equity between the partners is clearly outlining mutually agreed-upon protocols. This practice provides a road map of each partner’s expectations. For example, the process of disseminating results to the community and reporting results for publication and to sponsors was perceived as a considerable challenge (Israel et al. 1998; O’Fallon and Dearry 2002), and this was discussed at length. Community members unfamiliar with epidemiologic research expected results to appear during the process of data collection. Previous studies at Akwesasne have been very slow to report results, and this memory can affect recruitment for the current project. In contrast, researchers perceive that final analyses cannot be completed until the entire sample is collected, often a long process in a small community, and results should not be disseminated until “vetted” by the process of peer review.

We developed a protocol in which results are categorized as those concerning the individual and those concerning the community, which involves a system of checks and balances whereby research results are reviewed by members of the Akwesasne community before they become final. Results pertaining to individual participants such as tests of toxicant levels, results from cognitive and behavioral tests, and physical growth assessments (height and weight percentiles) are returned to individuals and their physicians, as appropriate, as soon as they are available and well before data collection ceases. This process produces immediate benefits to the participants because they can then act on the information provided to improve their health (McCauley et al. 2001; O’Fallon and Dearry 2002). The protocol also improves the community’s trust and belief that final results will be returned to the community while reducing pressure on the research partners to deliver final results.

A second aspect of the protocol guides communication of results relating to the community, for example, results pertaining to the relationships among variables of interest on the population level. The main problem historically has been the public release of results about the community to research sponsors, and to scientific journals and eventually the press before the participants and the community at Akwesasne are informed. The result was that the community learned about themselves from others, including groups who were potential adversaries in legal action (e.g*.*, polluting industries and government agencies that may be sued for hindering remediation). This route of communication is contrary to community culture and is an essentially disempowering process. A related problem was that researchers, when asking for community comment, often did so when the final report was complete and did not ask for input during the development of the analysis or the writing of the report.

We developed the Albany–Akwesasne Protocol to guide the distribution of results and to incorporate community input during the process of report writing (Table 1). Initially, science and community partners prioritize report writing in the context of the project’s specific aims. Community partners are invited to share writing duties, and a preliminary draft is developed by the writing team. The draft is presented to the partnering community group or groups, and later a meeting is held to discuss comments on all aspects of the work but especially regarding the accurate depiction of the community. We try to allow at least 2 weeks between transmittal of the draft and the discussion session to allow community partners to read and discuss the manuscript. Revision of the manuscript occurs with community group partners, and when all authors agree, it is submitted to a peer-reviewed journal. Because changes are often required before publication, the editor’s comments are conveyed to the community partners involved in modifying the text and responding to the editor. When the manuscript is accepted but before publication, the results are presented to the community and to study participants at a community meeting. This process is designed to ensure that *a*) the community learns about itself directly, *b*) the community has input before the manuscript is completed, and *c*) the research itself has received the stamp of peer-reviewed approval so that the results disseminated to the community-at-large are accurate and not insulting. The process also enables those community members who have made significant contributions to the report to be co-authors.

Besides the obvious benefits of a more informed analysis of the data by virtue of community input, the process teaches researchers important details of community culture that are likely to be helpful in understanding the social production of health disparities. It also familiarizes community members with the culture of science, including its epistemology and economics, leading to greater understanding and sustainable partnerships with scientists (Israel et al. 1998; O’Fallon and Dearry 2002; Wallerstein 1999). In addition, it builds capacity in the community to write scientific reports and grants of their own (Israel et al. 2001; Stevens and Hall 1998).

The Albany–Akwesasne protocol facilitates equity by allowing both partners to receive credit for their work, input, and assistance. The policy of including community members or organizations as authors of scientific papers acknowledges the value of local expertise and recognizes the merit of contributions that have been made throughout the research process, from identifying research questions of interest to providing feedback and interpretation on papers for publication (Israel et al. 2001; Wallerstein 1999).

### Two-way communication.

Much of the partnership relationship is based on frequent and open two-way communication that is equally privileged (Holkup et al. 2004). As the project has progressed, communication content has varied with the phase of the research. Strong and frequent internal and external communication is required to maintain the working relationship between the academic researchers, the local project personnel, and the community. Very frequent contact is needed to ensure that community project personnel stay in the loop regarding what is occurring with the academic research team and vice versa. The examples described previously illustrate how such communication is essential for development of locally appropriate research design, data collection instruments, recruitment, report writing, and dissemination (Israel et al. 1998; O’Fallon and Dearry 2002).

## The Potential of Partnership Research to Understand Health Disparities

Understanding the relationship between health disparities and social and physical environments involves a detailed, highly contextualized, and carefully nuanced analysis of the myriad factors that are included in the simple words “social and physical environments.” Creating a model that depicts these relationships and then operationalizing the model for hypothesis testing is a formidable challenge. We believe the task is impossible without integrating the detailed knowledge of community members with scientific research methodologies. The local knowledge and input of community members has facilitated the development and successful implementation of our research design, which includes a rigorous data collection protocol in a population that is already burdened with grave social, political, and economic challenges related to 500 years of genocidal policies and neglect.

Few studies have been conducted on the health effects of toxicants on adolescents or young adults and even fewer on Native American youth. The intimate knowledge of the community by the local project personnel is the basis for the successful recruitment of this age group. Community knowledge has been invaluable in the development of the questionnaires and instruments during the project. Through community collaboration, the project has been able to develop culturally sensitive, as well as culturally relevant, instruments that capture complex pathways of exposure. Because several pathways of exposure in this community are potentially linked to culturally based activities that are closely connected to Mohawk identity, building a trusting partnership in research is critical. The Mohawk community at Akwesasne has many good reasons to be distrustful of outsiders, in general, and academic researchers specifically. It is only through a collaborative framework based on relationships of respect that a detailed investigation of the role of the social and physical environments on toxicant exposure can occur.

### Mechanisms to reduce health disparities.

One of the most important contributions of partnership research is its potential to build capacity in the communities where the research takes place. The long history of colonialism, forced containment to reservations, and ongoing federal policies directed at Native Americans has had profound effects on Native communities. Such deeply entrenched social disparities at Akwesasne have contributed to the placement of polluting industries next door to the community, and, consequently, to pattern exposure to environmental toxicants. Access to employment and education is limited within the community. Community members are closely tied to the land where they live, which is part of their ancestral Iroquois territories, yet they have three industrial waste sites as their current neighbors. These factors are not easily changed and are connected to larger political and economic forces that can be linked to changes at the global level.

On the local level the work of the community will continue after the research is finished. Actions as well as policy decisions are needed to resolve the environmental and health issues. As the ATFE has reviewed the risk assessments that have been completed, it has become obvious that while frameworks for risk assessments have evolved over time, there remains a void in the assessment of health. The void is grounded in a definition of health within Mohawk society that differs remarkably from that of mainstream society. Not only must the physical health of an individual be considered but also what has become known as the emotional, mental, and spiritual being of the person. Considering only the physical part of the individual does not address the health and well-being of the individual; therefore, overall health is at greater risk. Without this consideration, any risk assessment is lacking and cannot address the very issue it is supposed to address. The definitions of health used by Tribal/First Nations are strikingly different from those of Western health-based professionals and scientists. Moreover, there is a critical need to expand the current definition of health and incorporate traditional knowledge into all facets of decision making regarding health issues.

The results of this project will provide part of the picture regarding risk of exposure and possible health effects in the community. Work is ongoing at Akwesasne to develop a more holistic model of risk-based decision making (Arquette et al. 2002). Results from this project will be integrated with information from many other community sources so that a full picture of the impact of toxicants in the community can be created. For the Mohawk community it is critical to identify correctly those cultural and subsistence-based pathways placing them at risk and then for them, as a community, to decide what is acceptable risk.

Research relationships with communities do not end when the funding does, as academic partners may be called on to assist with intervention and policy issues for years and perhaps decades into the future. Sustainability and reciprocity of the partnership relationship are the truest forms of benefit for the community (Holkup et al. 2004), for these will aid the community to reduce or effectively eliminate persistent racial and socioeconomic disparities in health in the future.

## Figures and Tables

**Table 1:** Albany–Akwesasne Protocol for dissemination of results.

Researchers and community partners discuss and choose hypotheses to be tested. Partners are invited to collaborate on writing. Development of preliminary draft. Draft manuscript is presented to the partnering community group or groups. Comments are received by authors concerning all aspects of the work, especially regarding the accurate depiction of the community. Writing partners revise the manuscript to joint satisfaction. Manuscript is submitted to a peer-reviewed journal. Comments from the editor are shared with partners; modifications to the manuscript and responses to the editor are constructed by partners. Upon acceptance and before publication, the results are presented to the community at large and study participants at a community meeting. Publication
